# Genome Sequence Analysis of *Exiguobacterium* sp. Strain TBG-PICH-001, Isolated from Pichavaram Mangrove Forest in South India

**DOI:** 10.1128/mra.00096-22

**Published:** 2022-04-18

**Authors:** Shiburaj Sugathan, Sajna Salim, Nitin Srivastava, Prakashkumar Ravindran, Sunil K. Khare

**Affiliations:** a Department of Botany, University of Kerala, Kariavattom Campus, Thiruvananthapuram, Kerala, India; b Department of Biotechnology, University of Kerala, Kariavattom Campus, Thiruvananthapuram, Kerala, India; c Enzyme and Microbial Biochemistry Laboratory, Department of Chemistry, Indian Institute of Technology Delhi, Hauz Khas, New Delhi, India; d Jawaharlal Nehru Tropical Botanic Garden and Research Institute, Palode, Thiruvananthapuram, Kerala, India; University of Rochester School of Medicine and Dentistry

## Abstract

The draft genome sequence of *Exiguobacterium* sp. strain TBG-PICH-001, isolated from Pichavaram Mangrove Forest (Tamil Nadu, India), is reported here. Paired-end sequencing technology was used to sequence the genome on the Illumina HiSeq X Ten platform. The genome comprises 3,141,454 bp; it harbors 3,154 genes and has a G+C content of 47.34%.

## ANNOUNCEMENT

*Exiguobacterium* is a genus of Gram-positive, low GC content, and non-spore-forming bacteria that belongs to the phylum *Firmicutes*. This genus is known for its incomparable diversity and tolerance to various extreme environments (1). *Exiguobacterium* sp. strain TBG-PICH-001 was isolated from a soil sample from Pichavaram Mangrove Forest (11.4226°N, 79.7748°E) (2). The world’s second-largest mangrove wetland, Pichavaram is situated on the southeastern coast of South India in the Cuddalore District of Tamil Nadu State (3). The bacterium was isolated following the dilution plate method, using skim milk agar plates containing casein hydrolysate (0.5%), yeast extract (0.25%), skimmed milk powder (1.0%), and agar (1.0%), with a pH of 7.0. The plates were incubated for 48 h at 30°C. Bacterial colonies with a clear zone around them indicating proteolytic activity were picked, made into a pure culture, and preserved as a glycerol suspension at −80°C in 25% glycerol. This isolate can produce alkaline protease (2) and is tolerant to organic solvents (4). Solvent-susceptible bacteria are instrumental in solubilizing various substrates in manufacturing different industrial end products used through nonaqueous biocatalysis (5).

For DNA extraction, a single colony of *Exiguobacterium* sp. TBG-PICH-001 was inoculated (10% vol/vol inoculum) into yeast extract-peptone (YP) broth containing 0.2% yeast extract, 0.5% peptone, and 2.5% NaCl and grown for about 48 h at room temperature (27 ± 1°C) on a shaker at 120 rpm.

**FIG 1:**
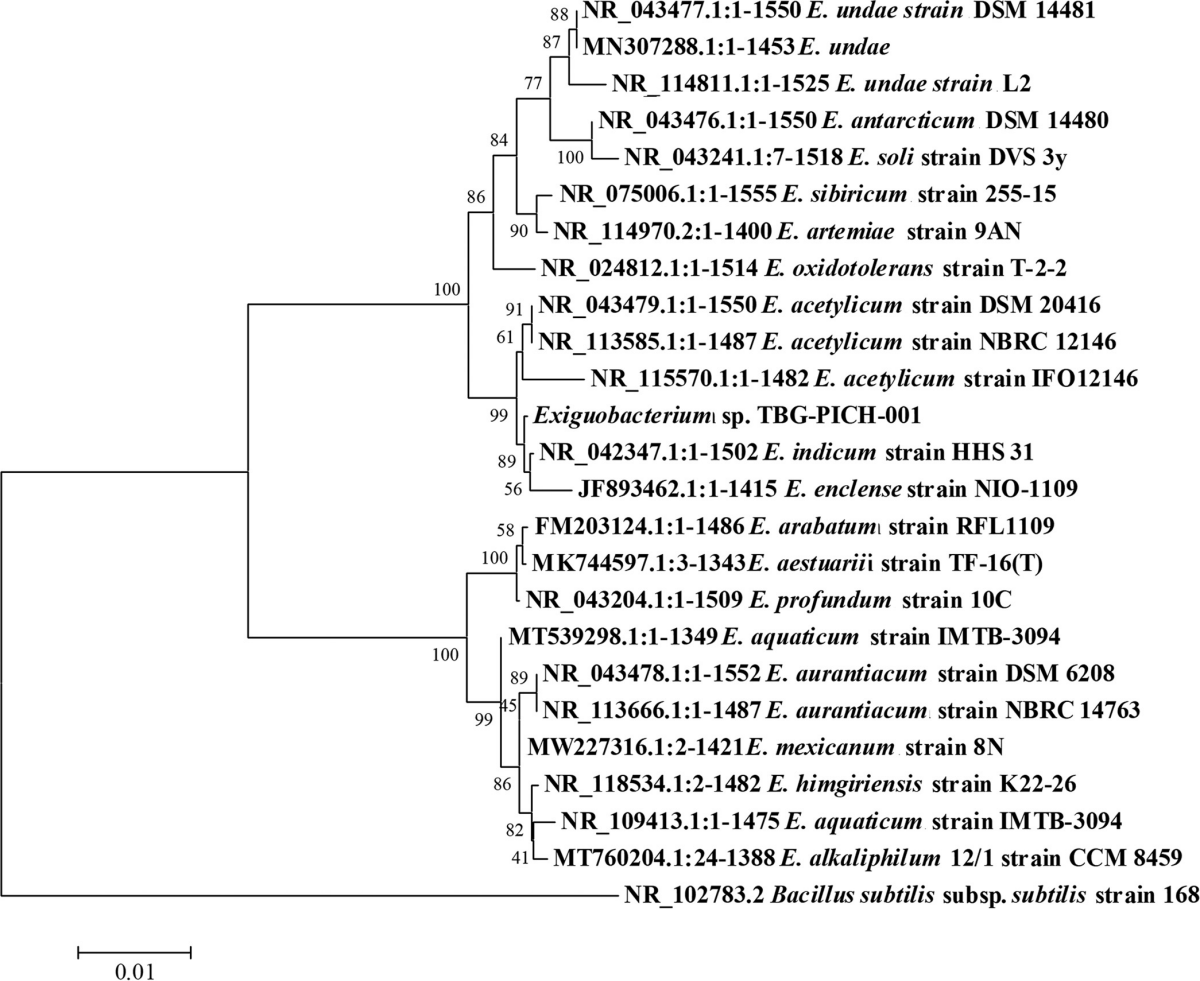
16S rRNA-based phylogenetic tree of *Exiguobacterium* sp. strain TBG-PICH-001 (drawn on 25 September 2021). The evolutionary history was inferred using the neighbor-joining method (12). The optimal tree is shown, with a branch length sum of 0.10987069. The percentage of replicate trees in which the associated taxa clustered together in the bootstrap test (1,000 replicates) is shown next to the branches. The tree is drawn to scale, with branch lengths in the same units as those of the evolutionary distances used to infer the phylogenetic tree. The evolutionary distances were computed using the Jukes-Cantor method (14) and are in units of the number of base substitutions per site. The analysis involved 25 nucleotide sequences. All positions containing gaps and missing data were eliminated. There were a total of 1,284 positions in the final data set. Evolutionary analyses were conducted using MEGA6 (15).

Genomic DNA was extracted using a Wizard genomic DNA purification kit (Promega, USA) following the manufacturer’s protocol. The extracted DNA was quantified using a Qubit 4 fluorometer (Thermo Fisher Scientific, MA, USA). Genome sequencing of *Exiguobacterium* sp. TBG-PICH-001 was performed at AgriGenome Labs Private Limited, Kakkanad, Kerala, India. A sequencing library was created using the TruSeq Nano DNA library prep kit (Nextera mate pair library prep kit), following the instructions of the manufacturer. Sequencing of the paired-end library was performed on the Illumina HiSeq X Ten platform, with a read length of 2 × 150 bp. A fastq quality check was performed for base quality score distributions, average base content per read, and GC distribution in the reads. The fastq files were preprocessed by trimming the Illumina adapter sequences using the Cutadapt v1.8 (6) program, followed by removing the low-quality bases with an average quality score of less than 30 in any of the paired-end reads. The duplicate reads were also removed using FastUniq (7). The preprocessing yielded 1,683,207 reads (after quality control). *De novo* assembly was performed using Velvet v1.2.10 (8) software to produce 17 contigs and an *N*_50_ value of 1,872,748 bp. A k-mer value of 79 was used for the Velvet assembly. The assembly resulted in a genome sequence of 3,141,454 bp with a genome coverage of 97.0× and a GC content of 47.33%. The presence of conserved genes in the assembled contigs was analyzed using BUSCO v2 (9), and the score was C: 98.60% (S: 98.60%, D: 0.0%), F: 0.0%, M: 1.4%, N: 148%. Default parameters were used for all software. The gene prediction and functional annotation using the NCBI Prokaryotic Genome Annotation Pipeline are available at GenBank (10). A total of 3,154 protein-coding sequences were identified (11). Strain TBG-PICH-001 had an average nucleotide identity (ANI) (http://enve-omics.ce.gatech.edu/ani/index) of 95.12% and 94.10% with the closest species, Exiguobacterium indicum and Exiguobacterium acetylicum, respectively. These values are much lower than the generally accepted species threshold level of ≥96%, indicating a possible new species (12). The gene sequence encoding the 16S rRNA of strain TBG-PICH-001 (GenBank accession number NZ_JADOYC010000011) and those of the closely related species were retrieved from GenBank and aligned using ClustalW; phylogenetic trees were constructed using MEGA6 (13). A 16S rRNA-based phylogenetic tree is shown in Fig. 1. A 16S rRNA BLAST analysis showed that *Exiguobacterium* sp. TBG-PICH-001 is closely related to *E. acetylicum* strain DSM 20416^T^ (JNIR00000000) and *E. indicum* strain HHS 31^T^ (MPSZ00000000), with 99.61% and 99.80% similarities, respectively. Genome-based taxonomic analysis was performed by uploading the genome sequence to the Type (Strain) Genome Server (TYGS), available at https://tygs.dsmz.de/ (14). The digital DNA-DNA hybridization (dDDH) values using formula d4 are 58.1% to *E. acetylicum* strain DSM 20416^T^ and 60.1% to *E. indicum* strain HHS 31^T^. The results show that strain *Exiguobacterium* sp. TBG-PICH-001 does not belong to any species found in the TYGS database and possibly is a new species. Determining the species of TBG-PICH-001 will be the subject of future investigations.

### Data availability.

The complete genome sequence of *Exiguobacterium* sp. TBG-PICH-001 is available at DDBJ/ENA/GenBank under the accession number JADOYC000000000. The BioSample and BioProject accession numbers are SAMN16686066 and PRJNA674981, respectively. The SRA accession number is SRR16094771.

## References

[1] White RA, III, Soles SA, Gavelis G, Gosselin E, Slater GF, Lim DSS, Leander B, Suttle CA. 2018. The complete genome and physiological analysis of the Eurythermal firmicute *Exiguobacterium chiriqhucha* strain RW2 isolated from a freshwater microbialite, widely adaptable to broad thermal, pH, and salinity ranges. Front Microbiol 9:3189. doi:10.3389/fmicb.2018.03189. 30671032 PMC6331483

[2] Swaroop SK, Jithin V, Jijeesh V, Gayathri V, Shiburaj S, Haridas M, Sabu A. 2017. Production and purification of alkaline protease from *Exiguobacterium indicum* TBG-PICH-001 isolated from soil samples of Pichavaram Estuary (Tamil Nadu). Indian J Geomarine Sci 47:580–586. http://nopr.niscair.res.in/handle/123456789/44126.

[3] Srivastava J, Farooqui A, Hussain SM. 2012. Vegetation history and salinity gradient during the last 3700 years in Pichavaram Estuary, India. J Earth Syst Sci 121:1229–1237. doi:10.1007/s12040-012-0215-5.

[4] Srivastava N, Kumar S, Shiburaj S, Gupta A, Khare SK. 2021. Cellular adaptation responses in a halotolerant *Exiguobacterium* exhibiting organic solvent tolerance with simultaneous protease production. Environ Technol Innovation 23:101803. doi:10.1016/j.eti.2021.101803.

[5] Gupta A, Khare SK. 2009. Enzymes from solvent-tolerant microbes: useful biocatalysts for non-aqueous enzymology. Crit Rev Biotechnol 29:44–54. doi:10.1080/07388550802688797. 19514902

[6] Meier-Kolthoff JP, Göker M. 2019. TYGS is an automated high-throughput platform for state-of-the-art genome-based taxonomy. Nat Commun 10:2182. doi:10.1038/s41467-019-10210-3. 31097708 PMC6522516

[7] Martin M. 2011. Cutadapt removes adapter sequences from high-throughput sequencing reads. EMBnet J 17:10–12. doi:10.14806/ej.17.1.200.

[8] Xu H, Luo X, Qian J, Pang X, Song J, Qian G, Chen J, Chen S. 2012. FastUniq: a fast de novo duplicates removal tool for paired short reads. PLoS One 7:e52249. doi:10.1371/journal.pone.0052249. 23284954 PMC3527383

[9] Zerbino DR, Birney E. 2008. Velvet: algorithms for de novo short read assembly using de Bruijn graphs. Genome Res 18:821–829. doi:10.1101/gr.074492.107. 18349386 PMC2336801

[10] Haft DH, DiCuccio M, Badretdin A, Brover V, Chetvernin V, O'Neill K, Li W, Chitsaz F, Derbyshire MK, Gonzales NR, Gwadz M, Lu F, Marchler GH, Song JS, Thanki N, Yamashita RA, Zheng C, Thibaud-Nissen F, Geer LY, Marchler-Bauer A, Pruitt KD. 2018. RefSeq: an update on prokaryotic genome annotation and curation. Nucleic Acids Res 46:D851–D860. doi:10.1093/nar/gkx1068. 29112715 PMC5753331

[11] Jukes TH, Cantor CR. 1969. Evolution of protein molecules, p 21–132. In Munro HN (ed), Mammalian protein metabolism. Academic Press, New York, NY. doi:10.1016/B978-1-4832-3211-9.50009-7.

[12] Simão FA, Waterhouse RM, Ioannidis P, Kriventseva EV, Zdobnov EM. 2015. BUSCO: assessing genome assembly and annotation completeness with single-copy orthologs. Bioinformatics 31:3210–3212. doi:10.1093/bioinformatics/btv351. 26059717

[13] Tamura K, Stecher G, Peterson D, Filipski A, Kumar S. 2013. MEGA6: Molecular Evolutionary Genetics Analysis version 6.0. Mol Biol Evol 30:2725–2729. doi:10.1093/molbev/mst197. 24132122 PMC3840312

[14] Salzberg S, Delcher A, Kasif S, White O. 1998. Microbial gene identification using interpolated Markov models. Nucleic Acids Res 26:544–548. doi:10.1093/nar/26.2.544. 9421513 PMC147303

[15] Saitou N, Nei M. 1987. The neighbor-joining method: a new method for reconstructing phylogenetic trees. Mol Biol Evol 4:406–425. doi:10.1093/oxfordjournals.molbev.a040454. 3447015

